# Targeted drug approvals in 2023: breakthroughs by the FDA and NMPA

**DOI:** 10.1038/s41392-024-01770-y

**Published:** 2024-02-20

**Authors:** Lang Zheng, Wenjing Wang, Qiu Sun

**Affiliations:** 1 Department of Biotherapy, Cancer Center and State Key Laboratory of Biotherapy, West China Hospital, Sichuan University, Chengdu, 610041 China; 2 West China Medical Publishers, West China Hospital, Sichuan University, Chengdu, 610041 China

**Keywords:** Drug discovery, Diseases

According to the official website of China’s National Medicines and Pharmaceutical Administration (NMPA), a total of 87 novel drugs were approved in China in 2023, with targeted drugs accounting for 67.8% of the total, amounting to 59 drugs (Fig. 1a; Table 1) [https://www.nmpa.gov.cn/yaopin/ypjgdt/index.html]. Notably, domestic innovation is flourishing, with five first-in-class drugs, including Glumetinib, a c-Met inhibitor from Haihe Biopharma; Leritrelvir, a 3CL protease inhibitor from Raynovent; Anaprazole, a proton pump inhibitor from Xuanzhu Biopharm; Pegol-Sihematide, an EPO drug from Hansoh Pharma; and Zuberitamab from BioRay Biopharmaceutical Co., Ltd. Additionally, the world’s first allosteric inhibitor targeting TYK2, Sotyktu (deucravacitinib), has been approved for the treatment of psoriasis and Selumetinib, a MEK inhibitor co-developed by AstraZeneca and Merck Sharp & Dohme (MSD), became the first approved drug in China for the treatment of neurofibromatosis type I (NF1). Beyond these drugs, the approval of novel drug types such as CAR-T cell products, siRNA, monoclonal antibodies, dual antibodies, and ADCs in China is a significant development in the country’s pharmaceutical industry (Fig. 1a). Notably, Equecabtagene autoleucel, jointly developed by IASO Biotherapeutics and Innovent Biologics, became the first approved BCMA-targeted CAR-T cell therapy product in China. Additionally, Inaticabtagene autoleucel developed by Juventas was the first approved CAR-T cell therapy product in the field of leukemia treatment in China. In parallel, the acceleration in the rate of new drug approvals in China is also noteworthy. Glofitamab, for example, was approved in China only five months after its approval in the United States. Furthermore, numerous clinical trials for novel drugs are currently underway in China, and it is anticipated that these new medications will soon bring significant benefits to Chinese patients.

**Fig. 1:**
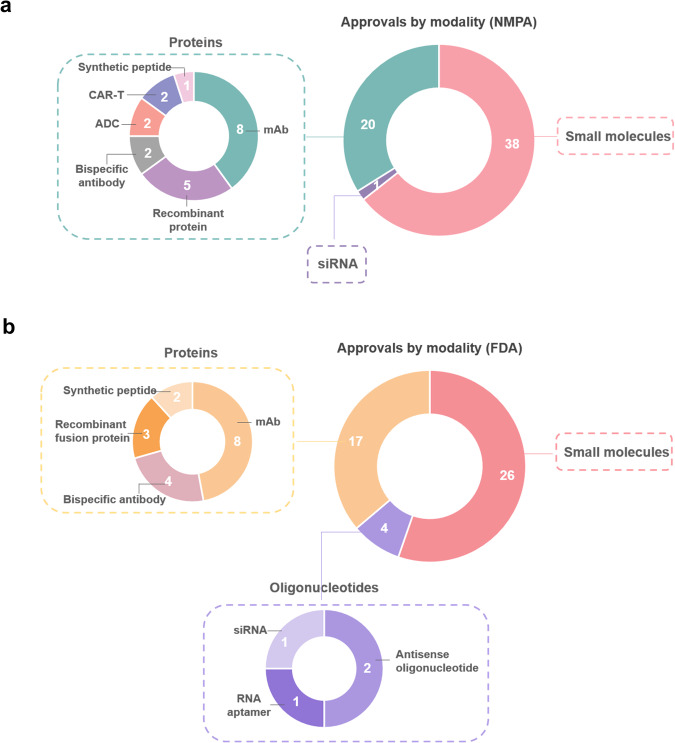
NMPA and FDA-approved targeted drugs by modality, 2023. **a** Small molecules and protein-based drugs comprised a significant majority of NMPA approvals, while oligonucleotides only represented a single case. **b** The majority of FDA approvals for targeted drugs consist of small molecules and protein-based drugs, with small molecules being approved as new molecular entities (NMEs). Approved in four cases, oligonucleotides were also classified as new molecular entities (NMEs). Source: NMPA, FDA and Nature Reviews Drug Discovery (10.1038/d41573-024-00001-x)

**Table 1:** Targeted drugs approved by the FDA and NMPA in 2023

No.	Brand name	Active ingredient	Approval date	Target/Activity	Approved use on approval date	Drug class	Company
FDA-approval							
1	Leqembi	Lecanemab-irmb	1/6/2023	Amyloid β (Aβ)	To treat Alzheimer’s disease	Monoclonal antibody (mAb)	Eisai, Biogen
2	Brenzavvy	Bexagliflozin	1/20/2023	Highly selective SGLT2 inhibitor	To enhance glycemic control in adults with type 2 diabetes, as a supplement to diet and exercise.	Small molecule	TheracosBio
3.	Jaypirca	Pirtobrutinib	1/27/2023	BTK inhibitor	To treat relapsed or refractory Mantle cell lymphoma (MCL) or Chronic lymphocytic leukemia (CLL).	Small molecule	Eli Lilly and Company
4	Orserdu	Elacestrant	1/27/2023	Estrogen receptor antagonist	To treat ESR1-mutated ER+/HER2- advanced or metastatic breast cancer.	Small molecule	Stemline Therapeutics
5	Jesduvroq	Daprodustat	2/1/2023	Reversible inhibitor of HIF-PH1, PH2 and PH3	To treat anemia that is caused by chronic kidney disease (CKD) in dialysis patients.	Small molecule	GlaxoSmithKline
6	Lamzede	Velmanase alfa-tycv	2/16/2023	Mannose-6-phosphate receptor	To treat non-central nervous system manifestations of alpha-mannosidosis	Recombinant fusion protein	Chiesi Farmaceutici S.p.A.
7	Filspari	Sparsentan	2/17/2023	ET_A_R and AT_1_R antagonist.	To lower protein in the urine (proteinuria) in adults with primary IgA nephropathy.	Small molecule	Travere Therapeutics, Inc.
8	Skyclarys	Omaveloxolone	2/28/2023	Nrf2 activator	To treat Friedrich’s ataxia	Small molecule	Reata Pharmaceuticals, Inc.
9	Zavzpret	Zavegepant	3/9/2023	Calcitonin gene-related peptide (CGRP) receptor antagonist	To treat migraine	Small molecule	Pfizer Inc.
10	Zynyz	Retifanlimab-dlwr	3/22/2023	PD-1	To treat metastatic or recurrent locally advanced Merkel cell carcinoma (MCC).	Monoclonal antibody (mAb)	Incyte Corporation
11	Joenja	Leniolisib	3/24/2023	PI3Kδ inhibitor	For the treatment of activated phosphoinositide 3-kinase delta (PI3Kδ) syndrome (APDS).	Small molecule	Pharming Group N.V.
12	Qalsody	Tofersen	4/25/2023	SOD1	To treat amyotrophic lateral sclerosis (ALS) with superoxide dismutase 1 (SOD1) mutation.	Gene therapy: antisense oligonucleotide (ASO)	Biogen MA Inc.
13	Veozah	Fezolinetant	5/12/2023	NK3 receptor antagonist	To reduce moderate to severe vasomotor symptoms (hot flashes and night sweats) caused by menopause	Small molecule	Astellas Pharma US, Inc.
14	Epkinly	Epcoritamab-bysp	5/19/2023	CD20, CD3	To treat diffuse large B-cell lymphoma (DLBCL) that returned or didn’t respond after 2 or more prior treatments	Bispecific antibody	Genmab US, Inc.
15	Xacduro	Sulbactam, Durlobactam	5/23/2023	β-lactamase inhibitor	To treat hospital-acquired bacterial pneumonia and ventilator-associated bacterial pneumonia caused by susceptible isolates of *Acinetobacter baumannii-calcoaceticus complex*	Small molecule	Entasis Therapeutics Ltd.
16	Paxlovid	Nirmatrelvir, Ritonavir	5/25/2023	Nirmatrelvir is a main protease inhibitor, ritonavir is an HIV-1 protease inhibitor and CYP3A inhibitor	To treat mild-to-moderate COVID-19	Small molecule	Pfizer Inc.
17	Inpefa	Sotagliflozin	5/26/2023	SGLT2 and SGLT1 inhibitor	To treat heart failure, type 2 diabetes mellitus, chronic kidney disease, and other cardiovascular risk factors	Small molecule	Lexicon Pharmaceuticals, Inc.
18	Columvi	Glofitamab-gxbm	6/15/2023	CD20, CD3	To treat relapsed or refractory diffuse large B-cell lymphoma (DLBCL)	Bispecific antibody	Genentech, Inc.
19	Litfulo	Ritlecitinib	6/23/2023	JAK3/TEC inhibitor	To treat severely patchy hair loss	Small molecule	Pfizer Inc.
20	Rystiggo	Rozanolixizumab-noli	6/26/2023	FcRn	To treat adults with generalized myasthenia gravis (gMG) who are acetylcholine receptor (anti-AChR) antibody positive or muscle-specific tyrosine kinase (anti-MuSK) antibody positive.	Monoclonal antibody (mAb)	UCB, Inc.
21	Ngenla	Somatrogon-ghla	6/27/2023	GH receptor	To treat pediatric Growth Hormone deficiency.	Recombinant fusion protein	Pfizer Inc.
22	Beyfortus	Nirsevimab-alip	7/17/2023	RSV F protein	To prevent respiratory syncytial virus (RSV) lower respiratory tract disease	Monoclonal antibody (mAb)	Sanofi Pasteur, Inc. and AstraZeneca AB
23	Vanflyta	Quizartinib	7/20/2023	FLT3 inhibitor	For patients with newly diagnosed FLT3-ITD positive acute myeloid leukemia (AML).	Small molecule	Daiichi Sankyo, Inc.
24	Xdemvy	Lotilaner	7/25/2023	Gamma-aminobutyric acid (GABA)-gated chloride channel inhibitor	To treat Demodex blepharitis	Small molecule	Tarsus Pharmaceuticals, Inc.
25	Zurzuvae	Zuranolone	8/4/2023	Gamma-aminobutyric acid (GABA) A receptor positive modulator	To treat postpartum depression	Small molecule	Sage Therapeutics, Inc., Biogen Inc.
26	Izervay	Avacincaptad pegol	8/4/2023	Complement protein C5 inhibitor	To treat geographic atrophy (GA).	Gene therapy: RNA aptamer	IVERIC bio, Inc.
27	Talvey	Talquetamab-tgvs	8/9/2023	CD3 receptor and G protein-coupled receptor class C group 5 member D (GPRC5D)	To treat adults with relapsed or refractory multiple myeloma who have received at least four prior therapies	Bispecific antibody	Janssen Biotech, Inc.
28	Elrexfio	Elranatamab-bcmm	8/14/2023	B-cell maturation antigen (BCMA) and CD3	To treat adults with relapsed or refractory multiple myeloma who have received at least four prior lines of therapy	Bispecific antibody	Pfizer, Inc.
29	Sohonos	Palovarotene	8/16/2023	RARγ agonist	To treat fibrodysplasia ossificans progressive.	Small molecule	Ipsen Biopharmaceuticals, Inc.
30	Veopoz	Pozelimab-bbfg	8/18/2023	Complement protein C5	To treat CD55-deficient protein-losing enteropathy, also known as CHAPLE disease.	Monoclonal antibody (mAb)	Regeneron Pharmaceuticals, Inc.
31	Aphexda	Motixafortide	9/8/2023	CXCR4 inhibitor	To use with filgrastim (G-CSF) to mobilize hematopoietic stem cells to the peripheral blood for collection and subsequent autologous transplantation in patients with multiple myeloma	Synthetic peptide analog	BioLineRx
32	Ojjaara	Momelotinib	9/15/2023	JAK1/JAK2, mutant JAK2^V617F^ and ACVR1 inhibitor	To treat anemia in adults with intermediate or high-risk myelofibrosis.	Small molecule	GlaxoSmithKline
33	Exxua	Gepirone	9/22/2023	5HT1A receptors agonists	To treat major depressive disorder	Small molecule	Mission Pharmacal Company
34	Rivfloza	Nedosiran	9/29/2023	lactate dehydrogenase A (LDHA)	To lower urinary oxalate levels in patients 9 years and older with primary hyperoxaluria type 1 and relatively preserved kidney function	Gene therapy: siRNA	Pyramid Laboratories, Inc.
35	Velsipity	Etrasimod	10/12/2023	Sphingosine 1-phosphate (S1P) receptor modulator	To treat moderately to severely active ulcerative colitis in adults	Small molecule	Pfizer Inc.
36	Zilbrysq	Zilucoplan	10/17/2023	Complement protein C5	To treat generalized myasthenia gravis (gMG) in patients who are AChR antibody positive .	Synthetic peptide analog	UCB, Inc.
37	Bimzelx	Bimekizumab	10/17/2023	Interleukin-17 A/F antagonist	To treat moderate to severe plaque psoriasis in adults who are candidates for systemic therapy or phototherapy	Monoclonal antibody (mAb)	UCB, Inc.
38	Agamree	Vamorolone	10/26/2023	Glucocorticoid receptor	To treat Duchenne muscular dystrophy	Small molecule	Santhera Pharmaceuticals (USA), Inc.
39	Omvoh	Mirikizumab-mrkz	10/26/2023	p19 subunit of IL-23	To treat ulcerative colitis	Monoclonal antibody (mAb)	Eli Lilly and Company
40	Loqtorzi	Toripalimab-tpzi	10/27/2023	PD-1	To treat advanced nasopharyngeal carcinoma (NPC).	Monoclonal antibody (mAb)	Coherus BioSciences, Inc.
41	Fruzaqla	Fruquintinib	11/8/2023	VEGFR1/2/3 inhibitor	To treat refractory, metastatic colorectal cancer	Small molecule	Takeda Pharmaceuticals America, Inc.
42	Augtyro	Repotrectinib	11/15/2023	ROS1, TRKA, TRKB, and TRKC inhibitor	To treat ROS1-positive non-small cell lung cancer	Small molecule	Bristol-Myers Squibb Company
43	Ryzneuta	Efbemalenograstim	11/16/2023	CSF-3R agonist	For the treatment of chemotherapy-induced neutropenia	Recombinant fusion protein	Evive Biotechnology Singapore PTE. Ltd.
44	Truqap	Capivasertib	11/16/2023	AKT1, AKT2 and AKT3 inhibitor	To treat breast cancer	Small molecule	AstraZeneca Pharmaceuticals LP
45	Ogsiveo	Nirogacestat	11/27/2023	Gamma secretase inhibitor	To treat adults with progressing desmoid tumors who require systemic treatment	Small molecule	SpringWorks Therapeutics, Inc.
46	Fabhalta	Iptacopan	12/5/2023	Complement Factor B inhibitor	To treat paroxysmal nocturnal hemoglobinuria	Small molecule	Novartis Pharmaceuticals Corporation
47	Wainua	Eplontersen	12/21/2023	TTR mRNA	To treat polyneuropathy of hereditary transthyretin-mediated amyloidosis	Gene therapy: antisense oligonucleotide (ASO)	AstraZeneca Pharmaceuticals LP
NMPA-approval							
1	Polivy	Polatuzumab Vedotin-Piiq	1/10/2023	CD79B	For the treatment of diffuse large B-cell lymphoma	ADC	Roche China Holding Ltd.
2	Exkivity	Mobocertinib	1/11/2023	EGFR inhibitor	For the treatment of adult patients with locally advanced or metastatic non-small cell lung cancer (NSCLC) that has progressed during or after platinum-containing chemotherapy and carries an insertion mutation in exon 20 of the epidermal growth factor receptor (EGFR).	Small molecule	Takeda Pharmaceutical Co., Ltd.
3	Kispali	Ribociclib	1/19/2023	CDK4/6 inhibitor	For the treatment of breast cancer	Small molecule	Beijing Novartis Pharma Ltd.
4		Simnotrelvir/Ritonavir	1/28/2023	HIV-1 protease, SARS-CoV 3CLpro inhibitor	For the treatment of adult patients with mild to moderate novel coronavirus infection (COVID-19)	Small molecule	Simcere Pharmaceutical (Hainan) Co., Ltd.
5		Deuremidevir	1/29/2023	RdRp inhibitor	For the treatment of adult patients with mild to moderate novel coronavirus infection (COVID-19)	Small molecule	Shanghai Wangshi Biomedical Technology Co., Ltd.
6	ZEPOSIA	Ozanimod	1/31/2023	S1PR1/5 regulator	For the treatment of multiple sclerosis	Small molecule	Bristol-Myers Squibb (China) Investment Co. Ltd.
7		Keverprazan	2/14/2023	Potassium-competitive acid blockers	For the treatment of duodenal ulcer and reflux oesophagitis	Small molecule	Jiangsu Carephar Pharmaceutical Co., Ltd.
8	Enhertu	Fam-trastuzumab deruxtecan-NXK	2/21/2023	HER2 antagonist	For the treatment of breast cancer	ADC	Daiichi Sankyo (China) Holdings Co., Ltd.
9		Adebrelimab	2/28/2023	PD-L1	For the first-line treatment of extensive-stage small cell lung cancer (ES-SCLC) in combination with chemotherapy	Monoclonal antibody (mAb)	Shanghai Shengdi Pharmaceutical Co., Ltd.
10		Glumetinib	3/08/2023	c-MET inhibitor	To treat locally advanced or metastatic non-small cell lung cancer with a mesenchymal-epithelial transforming factor (MET) exon 14 skipping mutation.	Small molecule	Haihe Biopharma Co., Ltd.
11	Calquence	Acalabrutinib	3/21/2023	BTK inhibitor	For the treatment of adult patients with metachronous lymphoma (MCL) who have received at least one prior therapy	Small molecule	AstraZeneca Investment (China) Co., Ltd.
12		Leritrelvir	3/23/2023	SARS-CoV-2 3CLpro inhibitor	For the treatment of adult patients with mild-to-moderate novel coronavirus infection (COVID-19)	Small molecule	Guangdong Raynovent Biotech Co., Ltd.
13	Koselugo	Selumetinib	4/28/2023	MEK1/2 inhibitor	For the treatment of neurofibromatosis type 1	Small molecule	AstraZeneca Investment (China) Co., Ltd.
14	Olinvyk	Oliceridine	4/28/2023	μ opioid agonist	For the treatment of acute pain	Small molecule	Jiangsu Nhwa Pharmaceutical Co., Ltd.
15	Ryzneuta	Efbemalenograstim alfa	5/06/2023	CSF-3R agonist	For the treatment of chemotherapy-induced neutropenia	Recombinant fusion protein	Evive Biopharmaceutical (Beijing) Ltd.
16		Alfosbuvir	5/12/2023	HCV NS5B RNA-dependent RNA polymerase inhibitor	For the treatment of primary or interferon-treated genotypes 1, 2, 3, and 6 chronic hepatitis C virus (HCV) infection in adults with or without compensated cirrhosis	Small molecule	Nanjing Sanhome Pharmaceutical Co., Ltd.
17		Zuberitamab	5/12/2023	CD20	For the treatment of CD20-positive diffuse large B-cell lymphoma (DLBCL)	Monoclonal antibody (mAb)	BioRay Biopharmaceutical Co., Ltd.
18	Aliqopa	Copanlisib	5/19/2023	PI3Kα/δ inhibitor	For adult patients with relapsed or refractory follicular lymphoma (FL) who have received at least two previous systemic treatments	Small molecule	Bayer Healthcare Co., Ltd.
19	ILUMYA	Tildrakizumab-ASMN	5/26/2023	IL-23p19	For the treatment of plaque psoriasis	Monoclonal antibody (mAb)	Shenzhen Kangzhe Biotech Co., Ltd.
20	LIVMARLI	Maralixibat Chloride	5/29/2023	IBAT inhibitor	For the treatment of Alagille syndrome	Small molecule	Beihai Kangcheng (Suzhou) Biopharmaceutical Co., Ltd.
21		Befotertinib	5/29/2023	EGFR T790M inhibitor	For the treatment of patients with locally advanced or metastatic non-small cell lung cancer who are positive for the EGFR T790M mutation	Small molecule	Betta Pharmaceuticals Co., Ltd.
22		Vorolanib	6/07/2023	Multi-target kinase (VEGFR2、KIT、PDGFR、FLT3 and RET) inhibitor	For the treatment of patients with advanced renal cell carcinoma who have failed prior tyrosine kinase inhibitor therapy	Small molecule	Betta Pharmaceuticals Co., Ltd.
23	Aligrin	Bilastine	6/21/2023	H1 receptor antagonist	For the treatment of urticaria in adolescents and adults aged 12 years and over	Small molecule	A. Menarini China Holding Co., Ltd.
24		Anaprazole	6/21/2023	Proton pump inhibitor	For the treatment of duodenal ulcers	Small molecule	Xuanzhu (Beijing) Pharmaceutical Technology Co., Ltd.
25		Iruplinalkib	6/27/2023	ALK inhibitor	For the treatment of locally advanced or metastatic non-small cell lung cancer (NSCLC) that is mesenchymal lymphoma kinase (ALK)-positive	Small molecule	Qilu Pharmaceutical Co., Ltd.
26		Retagliptin	6/27/2023	DPP-4 inhibitor	For improving glycaemic control in adults with type 2 diabetes	Small molecule	Jiangsu Hengrui Pharmaceuticals Co., Ltd.
27	Carbaglu	Carglumic acid	6/27/2023	CPS1 activator	For the treatment of hyperammonaemia	Small molecule	Recordati (Beijing) Pharmaceutical Co., Ltd
28	Vivjoa	Oteseconazole	6/27/2023	Fungal CYP51 inhibitor	For the treatment of severe vulvovaginal candidiasis (VVC)	Small molecule	eVenus Pharmaceutical Laboratories, Inc.
29	Mulpleta	Lusutrombopag	6/27/2023	TPO receptor agonist	For the treatment of chronic liver disease with thrombocytopenia	Small molecule	Suzhou Ceclor Pharmaceutical Co., Ltd.
30		Telpegfilgrastim	6/30/2023	CSF-3R agonist	For the treatment of febrile neutropenia	Recombinant protein	Xiamen Amoytop Biotech Co., Ltd.
31		Equecabtagene autoleucel	6/30/2023	B-cell maturation antigen (BCMA)	For the treatment of adult patients with relapsed or refractory multiple myeloma	CAR-T	Nanjing IASO Biopharmaceutical Co., Ltd.
32		Pegol-Sihematide	6/30/2023	EPO receptor agonists	For the treatment of anemia caused by chronic kidney disease	Synthetic peptide analog	Jiangsu Hansoh Pharmaceutical Co., Ltd.
33	Wakix	Pitolisant	6/30/2023	H3 receptor antagonist	For the treatment of narcolepsy	Small molecule	Citrine Pharmaceutical (Shanghai) Co., Ltd.
34	Remitch	Nalfurafine	6/30/2023	κ opioid receptor agonist	For the treatment of pruritus	Small molecule	Shenyang Sunshine Pharmaceutical Co., Ltd.
35	Vocabria	Cabotegravir	7/11/2023	HIV integrase inhibitor	For the treatment of HIV infection	Small molecule	GlaxoSmithKline (China) Investment Co., Ltd.
36	REZUROCK	Belumosudil	8/01/2023	ROCK1/2 inhibitor	For the treatment of chronic graft-versus-host disease	Small molecule	BioNova Pharmaceuticals (Shanghai) Ltd.
37		Tafolecimab	8/15/2023	PCSK9 inhibitor	For the treatment of primary hypercholesterolemia (including heterozygous familial and non-familial hypercholesterolemia) and mixed dyslipidemia	Monoclonal antibody (mAb)	Innovent Biologics (Suzhou) Co., Ltd.
38	Leqvio	Inclisiran	8/22/2023	PCSK9	For the treatment of hypercholesterolemia or mixed dyslipidemia	siRNA	Beijing Novartis Pharma Ltd.
39		Sunvozertinib	8/22/2023	EGFR tyrosine kinase inhibitor	For the treatment of adult patients with locally advanced or metastatic non-small cell lung cancer (NSCLC) with EGFR exon 20 insertion mutations	Small molecule	Dizal Pharmaceutical Co., Ltd.
40	MARGENZA	Margetuximab	8/29/2023	HER2 antagonist	For the treatment of HER2-positive breast cancer	Monoclonal antibody (mAb)	Zai Lab Ltd.
41		Narlumosbart	9/05/2023	RANKL inhibitor	For the treatment of adult patients with giant cell tumours of bone that are not surgically resectable or whose surgical resection may result in severe functional impairment	Monoclonal antibody (mAb)	Shanghai JMT Biological Technology Co., Ltd.
42	Lysodren	Mitotane	9/05/2023	Steroid hormone receptor antagonist	For the treatment of adrenocortical carcinoma	Small molecule	Jiedi Pharmaceutical Technology (Shanghai) Co., Ltd.
43	Nexviazyme	Avalglucosidase alfa	9/28/2023	α-glucosidase	For the treatment of glycogen storage disease type II	Recombinant protein	Sanofi (China) Investment Co., Ltd.
44	Sotyktu	Deucravacitinib	10/18/2023	TYK2 inhibitor	For adult patients with moderate to severe plaque psoriasis who are suitable for systemic therapy or phototherapy	Small molecule	Bristol-Myers Squibb Company
45	Litfulo	Ritlecitinib	10/19/2023	JAK3/TEC inhibitor	To treat severely patchy hair loss	Small molecule	Pfizer Inc.
46	Kineret	Anakinra	10/27/2023	IL1R1	For the treatment of periodic fever syndrome	Recombinant protein	Supi Pharmaceutical (Shanghai) Co., Ltd.
47		Aponermin	11/01/2023	DR4/DR5 agonist	For the treatment of relapsed or refractory multiple myeloma	Recombinant protein	Wuhan Hiteck Biological Pharma Co., Ltd.
48	Columvi	Glofitamab	11/07/2023	CD20, CD3	For the treatment of adult patients with relapsed or refractory diffuse large B-cell lymphoma who have received at least two or more lines of systemic therapy	Bispecific antibody	Roche Pharma (Schweiz) AG
49		Inaticabtagene autoleucel	11/07/2023	CD19	For the treatment of relapsed or refractory B-cell acute lymphoblastic leukaemia in adults.	CAR-T	Juventas Cell Therapy Ltd.
50		Vebreltinib	11/14/2023	c-Met inhibitor	For the treatment of locally advanced or metastatic non-small cell lung cancer	Small molecule	Beijing Purunao Biotechnology Co., Ltd.
51	XENLETA	Lefamulin	11/14/2023	50S subunit	For the treatment of community acquired pneumonia	Small molecule	Sumitomo Pharmaceuticals (Suzhou) Co., Ltd.
52	Adasuve	Loxapine	11/21/2023	5-HT2A receptor, D2 receptor antagonist	For the treatment of adult schizophrenia or bipolar I disorder	Small molecule	Zhaoke Pharmaceutical (Hefei) Co., Ltd.
53		Atilotrelvir/Ritonavir	11/24/2023	HIV-1 protease, SARS-CoV-2 3CLpro inhibitor	For the treatment of COVID-19	Small molecule	Fujian Guangsheng Zhonglin Biotechnology Co., Ltd.
54		Dimdazenil	11/28/2023	GABAA receptor positive allosteric modulator	Short-term treatment for patients with insomnia	Small molecule	Zhejiang Jingxin Pharmaceutical Co., Ltd.
55	Tepmetko	Tepotinib	12/05/2023	c-Met inhibitor	For the treatment of non-small cell lung cancer	Small molecule	Merck Serono (Beijing) Pharmaceutical R&D Co., Ltd.
56	Vabysmo	Faricimab	12/13/2023	Ang2, VEGF-A	For the treatment of diabetic macular oedema	Bispecific antibody	Roche China Holding Ltd.
57	Livtencity	Maribavir	12/19/2023	UL97 inhibitor	For the treatment of cytomegalovirus infections	Small molecule	Takeda (China) International Trading Co., Ltd.
58		Socazolimab	12/19/2023	PD-L1	For the treatment of uterine cervical cancer	Monoclonal antibody (mAb)	Zhaoke (Guangzhou) Oncology Pharmaceutical Ltd.
59	Beyfortus	Nirsevimab	12/26/2023	RSV F protein	To prevent respiratory syncytial virus (RSV) lower respiratory tract disease	Monoclonal antibody (mAb)	AstraZeneca Investment (China) Co., Ltd.

During the year, the FDA’s Centre for Drug Evaluation and Research (CDER) approved 55 novel drugs, surpassing 37 in 2022 and second only to 59 in 2018^1^, [https://www.fda.gov/drugs/new-drugs-fda-cders-new-molecular-entities-and-new-therapeutic-biological-products/novel-drug-approvals-2023]. Of these novel drugs, 47, or 85.5%, were targeted therapies, reflecting a clear trend towards more personalized and effective treatment options. Within this category, 26 were small molecules, 8 were monoclonal antibodies, 4 were bispecific antibodies, 3 were recombinant fusion proteins, 4 were small nucleic acid drugs (including 2 antisense oligonucleotides, 1 RNA aptamer, and 1 siRNA), and 2 were synthetic peptide analogs (Fig. 1b; Table 1). The targets of these new drugs span a range of biological processes, including kinases, cytokines, enzymes, receptors, ion channels, and proteasomes. This diversity highlights the ongoing efforts to develop innovative therapies that target the root causes of various diseases. In terms of indications approved for marketing, cancer remains the leading focus of research and development, with 14 new cancer therapies, representing 29.7% of the total. Immune system disorders followed closely behind, accounting for 8 approved therapies (17%) while neurological disorders ranked third, with 7 approved therapies (14.9%). Diseases of the blood and lymphatic system, endocrine and metabolic diseases, and infectious diseases each accounted for four cases (8.5%) (Fig. 1b). This distribution highlights the diverse range of therapeutic areas targeted by novel drug development. Notably, there has been a gradual increase in targeted therapeutics for rare diseases, such as Pompe’s disease and paroxysmal sleep hemoglobinuria. These diseases often lack effective treatment options, making the development of targeted therapies particularly important for improving patient outcomes. Overall, the data presented in this article highlights the significant progress being made in the field of drug development, with a particular emphasis on targeted therapies. The increasing approval of novel drugs offers hope to patients with various diseases, and further research and development is expected to lead to even more effective and personalized treatment options in the future.

It is heartening to observe that the FDA has granted approved three innovative Chinese new drugs: Loqtorzi (toripalimab), Fruzaqla (fruquintinib) and Ryzneuta (efbemalenograstim). Toripalimab is a monoclonal antibody targeting PD-1, marks the first FDA-approved drug for the treatment of nasopharyngeal cancer. Fruquintinib, approved for the treatment of metastatic colorectal cancer, is unique as the first and only highly selective inhibitor approved in the U.S. for comprehensive inhibition of all three VEGF receptor kinases, regardless of a patient’s biomarker status. Efbemalenograstim, approved for the treatment of neutropenia in oncology patients receiving anti-cancer drugs, stands out as the only long-acting G-CSF (granulocyte colony-stimulating factor) product approved in both China and the U.S., showcasing its global relevance and potential impact on patient care^2^. As a result, Yifan Pharmaceutical Co., Ltd. has become the first innovative biopharmaceutical company in China to be approved by the FDA as a Marketed Access Holder (MAH), marking a significant milestone in the company’s journey towards global leadership in biopharmaceutical innovation.

Innovation in mechanism-based therapies reached new heights in the past year, with numerous drugs earning the distinction of “First-In-Class". These groundbreaking therapies either represent the first treatment option for a specific disease or introduce a novel mechanism of action. One remarkable example is Daprodustat, the first oral drug approved by the FDA for the treatment of anemia with chronic kidney disease (CKD) in dialysis patients^3^. Daprodustat reversibly inhibits HIF-PH1/2/3, thereby increasing HIF levels. This, in turn, stimulates the expression of genes essential for red blood cell production, such as EPO and VEGF. Another notable development is the approval of Fezolinetant, the world’s first non-hormone targeted drug, for the treatment of moderate to severe hot flashes caused by menopause. Unlike hormonal drugs commonly used to manage these symptoms, Fezolinetant works primarily by antagonizing the neurokinin-3 (NK3) receptor which offers patients a better quality of life with fewer side effects and more pronounced therapeutic effects.

Furthermore, 2023 marked a significant milestone in gene therapy with the approval of four groundbreaking nucleic acid drugs. Among them, Qalsody (tofersen) stands out as the first and currently only gene therapy designed to target the underlying pathogenesis of amyotrophic lateral sclerosis (ALS). Tofersen, an antisense oligonucleotide (ASO), specifically targets mRNA produced by the SOD1 mutant gene, halting the production of the toxic SOD1 protein and slowing the progression of ALS. Another noteworthy ASO therapy, Wainua (eplantersen), has been approved for marketing to inhibit the production of the TTR protein for the treatment of both hereditary and non-hereditary amyloidosis polyneuropathy. This innovative approach utilizes antisense oligonucleotide ligand coupling (LICA) technology, coupling ASO drugs to ligand molecules that bind to specific receptors on the cell surface. In addition, Izervay (avacincaptad pegol) represents a novel complement C5 protein inhibitor and the second FDA-approved RNA aptamer^4^. The approval of Avacincaptad pegol signifies the emergence of a potentially transformative innovator in the field of Geographic Atrophy (GA). Lastly, Rivfloza (nedosiran) was approved by the FDA in September 2023 as the second siRNA drug worldwide for the treatment of primary hyperoxaluria type 1 (PH1). This latest development showcases the increasing impact of gene therapy in treating previously intractable diseases and offers new hope for patients suffering from debilitating conditions.

In the realm of antibody therapeutics, we have witnessed the emergence of four groundbreaking bispecific antibodies capable of binding to two distinct epitopes or antigens simultaneously. This unique dual-action mechanism enhances target specificity, paving the way for innovative immunotherapy advancements. Notable examples include the Epcoritamab and Glofitamab, which target both CD20 and CD3, showcasing the potential of this approach in treating a range of diseases.

Targeted therapies continue to constitute the majority of approved therapies worldwide. However, immunotherapy has emerged as a significant avenue in cancer treatment in recent years, particularly when combined with targeted therapies, leading tosuperior therapeutic outcomes. This combination intervenes in the tumor’s immune escape mechanisms, thereby enhancing the immune cells’ attack capabilities and ultimately enhancing the efficacy of immunotherapy. Future advancements in targeted therapies will involve the identification and development of novel targets that specifically target immune escape mechanisms and interfere with immune cell-tumor interactions to improve immunotherapy effectiveness. Furthermore, the utilization of technologies such as RNA interference, gene editing, and other intracellular targeting methods will enable more precise therapeutic strategies.

In 2024, Signal Transduction and Targeted Therapy will continue to push the boundaries of targeted therapies, introducing innovative and disease-focused solutions. With a strong focus on clinical research, our aim is to publish more groundbreaking papers on novel therapeutic targets, signaling pathways, and effective new therapies especially drug discovery with clinical applications.

## References

[1] Mullard, A. 2023 FDA approvals. *Nat. Rev. Drug Discov.*10.1038/d41573-024-00001-x (2024). 10.1038/d41573-024-00001-x 38168801

[2] Blair HA (2023). Efbemalenograstim alfa: first approval. Drugs.

[3] Allison, S. J. Daprodustat for anaemia in CKD. *Nat. Rev. Nephrol*. **18**, 3 (2022). 10.1038/s41581-021-00515-2 34824467

[4] Kang C (2023). Avacincaptad pegol: first approval. Drugs.

